# The Impact of Early Therapeutic Intervention on the Central Pattern Generator in Premature Newborns – a Preliminary Study and Literature Review

**DOI:** 10.34763/devperiodmed.20192303.178183

**Published:** 2019-10-27

**Authors:** Magdalena Czajkowska, Anna Fonfara, Barbara Królak-Olejnik, Marcin Michnikowski, Tomasz Gólczewski

**Affiliations:** 1 Department of Neonatology, Wroclaw Medical University, Wroclaw, Poland; 2 Nałęcz Institute of Biocybernetics and Biomedical Engineering, Polish Academy of Sciences, Warsaw, Poland

**Keywords:** central pattern generator, premature newborn, sucking, Vojta method, centralny generator wzorca, wcześniak, ssanie, metoda Vojty

## Abstract

**The aim:**

To study the effect of therapeutic intervention on the improvement of the rhythmicity of non-nutritive and nutritive sucking in premature newborns and on the suck central pattern generator.

**Material and methods:**

Stimulation of the breast zone was performed in two premature newborns by means of the Vojta method. Intraoral pressure was measured during non-nutritive and nutritive sucking before and after this therapeutic intervention. The maximum negative pressures generated during individual sucks and the intervals between sucks were analysed.

**Results:**

The stimulation of the breast zone using the Vojta method seems to have no impact on the duration of individual sucking episodes. However, a significant improvement in the rhythmicity and regularity of sucking was observed in both newborns: the coefficient of quartile deviation for the intervals decreased from 15% and 11% to 13% and 6%, respectively, and for the maximum negative pressures it decreased from 24% and 27% to 9% and 19%, respectively. Additionally, the median value of the maximum negative pressure decreased in both newborns: from -39 and -37 mmHg to -45 and -60 mmHg, respectively.

**Conclusions:**

The stimulation of the breast zone using the Vojta method seems to have a direct impact on the central pattern generator, which improves the rhythmicity as well as the regularity of both non-nutritive and nutritive sucking.

## Streszczenie

**Cel:**

Badanie wpływu interwencji terapeutycznej na poprawę rytmiczności ssania nieodżywczego i ssania odżywczego u wcześniaków oraz na centralny generator wzorca ssania.

**Materiał i metody:**

Stymulację strefy piersiowej przeprowadzono u dwóch wcześniaków za pomocą metody Vojty. Zostało zmierzone wewnątrzustne ciśnienie podczas ssania nieodżywczego oraz ssania odżywczego przed i po interwencji terapeutycznej. Analizowano maksymalne podciśnienie w poszczególnych ssaniach i odstępy pomiędzy poszczególnymi zassaniami.

**Wyniki:**

Stymulacja strefy piersiowej wg metody Vojty wydaje się nie mieć wpływu na czas trwania poszczególnych epizodów ssania. Jednakże, znaczną poprawę rytmu i regularności ssania zaobserwowano w przypadku obu wcześniaków: współczynnik zmienności odstępów pomiędzy poszczególnymi zassaniami zmniejszył się z 15% i 11% do 13% i 6%, odpowiednio; a dla maksymalnego podciśnienia zmniejszył się z 24% i 27% do 9% i 19%. Dodatkowo, wartość mediany maksymalnego podciśnienia zmniejszyła się u obu wcześniaków: z -39 i -37 mmHg do -45 i -60 mmHg, odpowiednio.

**Wnioski:**

Stymulacja strefy piersiowej wg metody Vojty wydaje się mieć bezpośredni wpływ na centralny generator wzorca, co poprawia zarówno rytmiczność, jak i regularność ssania nieodżywczego oraz ssania odżywczego.

## Introduction

The slow development of premature newborn oral feeding skills is one of the main reasons for their prolonged stay in the neonatal unit. The transition from enteral to oral feeding is gradual and depends on the baby’s postmenstrual age (PMA), on the clinical condition of the infant and, in many cases, on therapeutic intervention.

The normal rhythmicity of the sucking, swallowing and breathing sequence is a direct indicator of effective feeding in premature newborns [1]. A theoretical model of the reflex neuromuscular control of sucking, swallowing and breathing refers to the concept of the central pattern generator (CPG), i.e. the distribution of neural networks and presumed microcircuits located near the pons and the spinal regions of the grey matter of the brainstem [2]. Amaizu et al. hypothesised that difficulties in premature newborn oral feeding result from dissimilar growth rates for different CPG parts, i.e. the sucking CPG (sCPG), the swallow CPG (wCPG), and the breath CPG (bCPG). The rates have an influence on the synchronisation of muscles operating in a specific CPG part and the coordination process of sCPG, wCPG and bCPG [3]. Mayerl et al. showed that prematurity impairs the development of coordination abilities, even though it does not affect the growth of the individual motions of sucking, swallowing and breathing [4].

Finan and Barlow suggest that sCPG reacts to the mechanical stimulation of soft tissue in the oral cavity and intraoral muscles [5]. Fucile et al. showed that 15-minute sensorimotor oral intervention strengthens and improves the coordination of sucking and swallowing, and thus has an impact on the neural development of sCPG. A 30-minute oral sensorimotor intervention increases the efficiency, volume and rate of feeding, and thus improves the coordination of sucking, swallowing and breathing controlled by CPG [6, 7, 8]. Randomised studies by Roch confirm that sensory stimulation accelerates the development of non-nutritive sucking in newborns with a very low birth weight [9]. Poore et al. also confirm that sensorimotor intervention effectively accelerates the development of sCPG in premature newborns [10] A study performed by Medoff-Cooper et al. enforces these observations, though in this case the visual, auditory and motor intervention on the sCPG was deployed [11].

The neurodevelopmental Vojta method [12] is considered a useful therapeutic intervention in newborns. For example, some research works on the Vojta therapy in premature newborns reported therapeutic effects on blood saturation and respiratory conditions within the framework of the respiratory therapy in the neonatal ward [13, 14]. It is of note that the therapy performed by trained staff is a noninvasive method which results in neither discomfort nor pain, i.e., correctly performed stimulation corresponds with the minimal handling policy.

To our knowledge, there were no previous studies related to the influence of the therapy on the rhythmicity and regularity of non-nutritive (NNS) and nutritive sucking (NS), and thus on the work of sCPG. This study compares NNS and NS before and after breast zone stimulation based on the Vojta method. The data shown are preliminary and come from an ongoing project analysing sucking in premature newborns.

## Aim

To study the effect of therapeutic intervention on the improvement of the rhythmicity of non-nutritive and nutritive sucking in premature newborns and on the suck central pattern generator.

## Material and methods

The study involved two premature newborns in the quiet alert state (readiness to suck). The newborns breathed on their own and no oxygen therapy was applied. Both premature newborns were fed with their mothers’ milk, however, with the use of a bottle teat. The age and weight of the newborns are given in Table I.

**Table I:** Characteristics of the newborns. Tabela I. Charakterystyka noworodków.

	Newborn 1 (NNS) *Noworodek 1 (ssanie nieodżywcze)*	Newborn 2 (NS ) *Noworodek 2. (ssanie odżywcze)*
gestational age [weeks] *wiek płodowy [tygodnie]*	29 2/7	25 0/7
birth weight [grams] *masa urodzeniowa [gramy]*	1.600	850
postmenstrual age on the day of measurement [weeks] *wiek postkoncepcyjny w dniu badania [tygodnie]*	33 0/7	34 5/7
weight on the day of measurement [grams] *masa w dniu badania [gramy]*	1.785	1.470

Newborn 1 was given a pacifier and was NNS-tested for 2 minutes. The test was performed in an incubator. The baby lay flat on its back. Without changing the position of the baby, the breast zone was stimulated based on the Vojta method. Sensory stimulation – a gentle touch – affects the skin of the intercostal area and lasts about 20 seconds alternately on each side of the chest. In total, the stimulation lasted 1 minute and 20 seconds. Following the therapy, the baby was directly positioned on a nursing pillow. At 2 minutes from the end of the therapy, another non-nutritive sucking measurement was performed on the same pacifier.

Newborn 2 was NS-tested for 3 minutes and 15 seconds during the morning bottle feeding. The test was performed in an incubator. Before the next feeding (3 hours later), the breast zone was stimulated based on the Vojta method. In total, the stimulation lasted 1 minute and 20 seconds. After the therapy, the baby was evaluated for nutritive sucking lasting 3 minutes and 15 seconds during bottle feeding.

The negative intraoral pressure during sucking was measured with a digital manometer designed and adjusted to this type of measurements at the Nałęcz Institute of Biocybernetics and Biomedical Engineering, Polish Academy of Sciences. The main components of the manometer are the Smiths Medical DPT-8003 pressure transmitter and commercial integrated circuits: AD7730, Atmega8A and FT230XS. The pressure gauge was connected to the oral cavity by means of an air-filled catheter attached to the nipple. The pressure was sampled at 25 Hz and recorded on a PC.

The effect of the breast zone stimulation on the maximum negative pressures generated during particular sucks and intervals between those sucks was analysed. The pressures and intervals were characterised by means of the lowest and median values, the quartile deviation and the quartile deviation coefficient.

The Bioethics Committee of the Wroclaw Medical University (No. KB-333/2018) approved the study. Each mother gave her oral and written consent to using stimulation and to monitoring NNS and NS measurements using a pressure gauge.

## Results

**Fig. 1:**
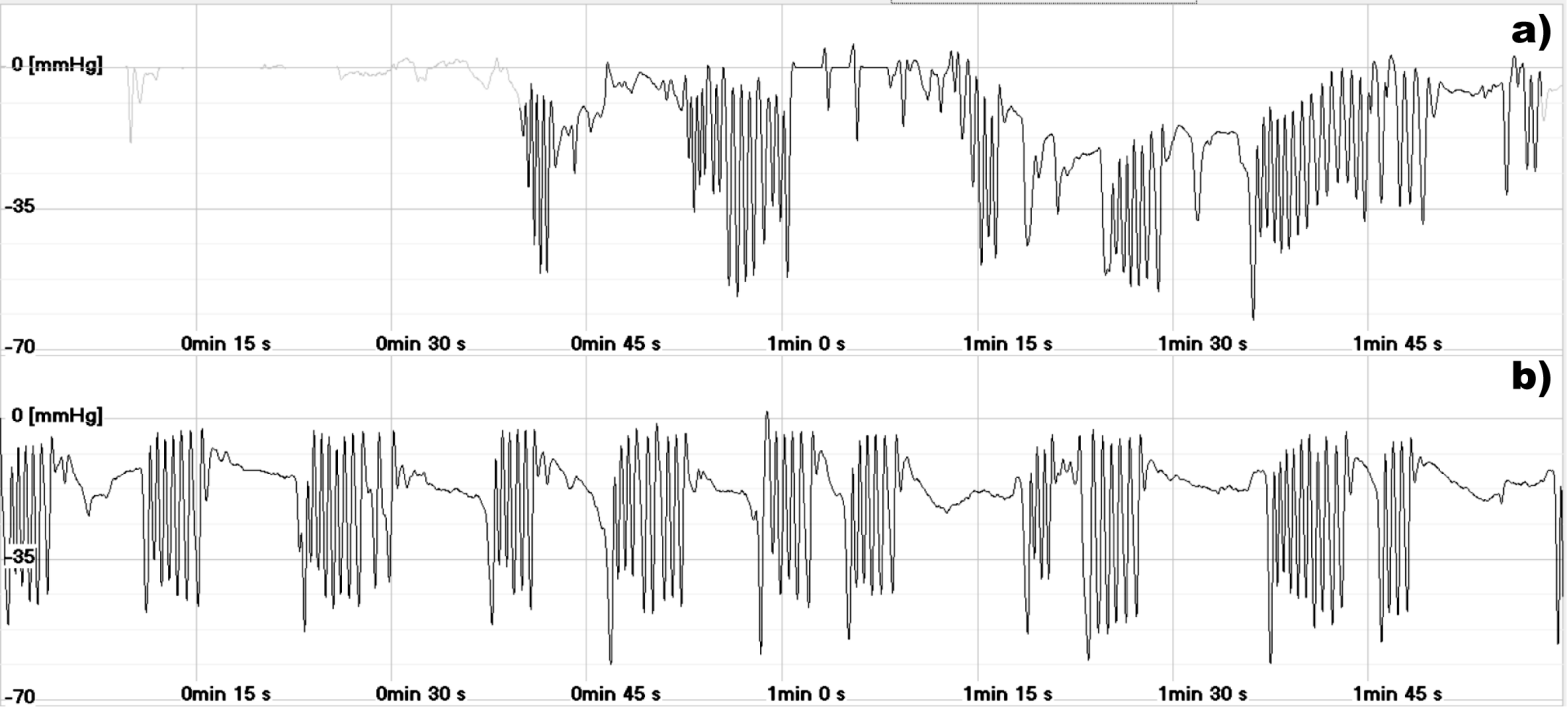
The intraoral pressure during non-nutritive sucking. a) before stimulation; b) after stimulation.

**Fig. 2:**
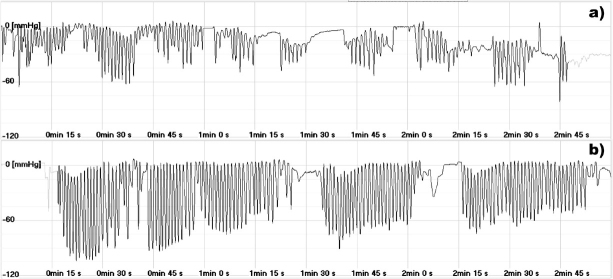
The intraoral pressure during nutritive sucking. a) before stimulation; b) after stimulation.

Figs. 1 and 2 present the pressure waveforms during sucking. Stimulation of the breast zone based on the Vojta method did not seem to affect the intervals between sucks. However, a significant improvement in rhythmicity, resulting in a decrease in both the quartile deviation and the coefficient of quartile deviation, was observed in both premature newborns (Tab. II). In the case of the maximum negative pressure, a decrease in the median and a significant decrease in the coefficient of quartile deviation were noted for both newborns (Tab. II).

**Table II:** Values of the parameters analysed before and after stimulation. Tabela II. Wartości analizowanych parametrów przed I po stymulacji.

Parameters *Parametry*	Newborn 1. *Noworodek 1*.		Newborn 2. *Noworodek 2*.	
	Before stimulation *Przed stymulacją*	After stimulation *Po stymulacji*	Before stimulation *Przed stymulacją*	After stimulation *Po stymulacji*
**Maximum negative pressure in particular sucks (Pmax) [mmHg]** ***Maksymalne podciśnienie w poszczególnych ssaniach [mmHg]***				
the lowest pressure *największe*	-62	-61	-80	-104
median *mediana*	-39	-45	-37	-60
quartile deviation *rozrzut*	9.7	4.0	9.7	11.6
coefficient of quartile deviation [%] *współczynnik zmienności [%]*	24	9	27	19
**Intervals between sucks [seconds]** ***Długość pojedynczych ssań [sekundy]***				
the lowest value *najkrótsze*	0.40	0.48	0.64	0.56
median *mediana*	0.64	0.64	1.12	1.04
quartile deviation *rozrzut*	0.10	0.08	0.12	0.06
coefficient of quartile deviation [%] *współczynnik zmienności [%]*	15	13	11	6

## Discussion

Stimulation based on the Vojta method seems to improve the rhythmicity and regularity of sucking in both NNS and NS. This result may suggest an effect on the sCPG which exercises neuromotor reflex control over muscles responsible for the sucking motions of a premature newborn. Most interestingly, even a single stimulation provided beneficial results.

The sucking motions of a premature newborn are generated by the coordinated action of muscle fibres of at least 26 muscle pairs and 5 pairs of cranial nerves, including the trigeminal nerve (V), the facial nerve (VII), the glossopharyngeal nerve (IX), the vagus nerve (X), the hypoglossal nerve (XII) and the cervical and thoracic segments of the spinal cord involved in the chest wall motions necessary to coordinate breathing and feeding [15, 16]. There is a theoretical model explaining how the sensory and motor nuclei as well as the sensory and motor pathways of these nerves are controlled at the level of the brainstem to perform rhythmic sucking motions in instinctive terms. Cyclic reflex movement patterns (e.g. walking, breathing, swallowing, sucking) are triggered by central pattern generators which consist of specialised networks of interneurons [17]. Sensorimotor control over oral feeding (sucking, swallowing and breathing) is coordinated by sCPG [9].

On each side, sCPG consists of a circuit of association neurons (interneurons) located in the reticular substance of the brainstem [5, 18]. Animal studies showed that rhythmic motor movements of the oral cavity can be triggered by the neural network which was proved to be located in the section of the pons between the trigeminal nerve nucleus and the facial nerve nucleus [19, 20]. Interneurons which form circuits responsible for rhythmic movements of the oral cavity have the internal ability to generate the bursts of sucking which are tonically inhibited in lower brainstem areas [21, 22]. In response to the central nervous system (CNS) and sensory signals [23], the sCPG changes the duration of the sucking cycle as well as the relevant duration and intensity of motor neuron bursts.

Barlow suggests that NS and NS represent the complex sensorimotor behaviour that can provide valuable information on the integrity of the CNS of a premature newborn [2]. He believes that sCPG located in the brainstem responds to the peripheral input of a sensory stimulus [24] and adapts to changes in the oral cavity [2, 5]. His conclusions are based on what he experienced when using the NTrainer biodevice. The device stimulates mechanoreceptors of the oral cavity muscles by means of the sensory stimulus [25, 26]. Fucile et al. applied touch and kinaesthetic stimulation of the oral cavity, face, torso and limbs of a premature newborn, and suggested that this kind of sensorimotor intervention affects the neural development of sCPG [7, 8]. This type of intervention also used a peripheral input, i.e. the sensory receptors on the newborn’s skin.

For the purpose of the peripheral input, the sensory stimulation based on Vaclav Vojta’s assumptions was applied. The breast zone, i.e. the area of the baby’s body located in the intercostal space of the chest (between the seventh and eighth rib) below the nipples, was stimulated. As a result of mechanical stretching of intercostal muscle fibres (belonging to the respiratory muscles), the intercostal nerves supplying these muscle groups and the phrenic nerve were activated under the influence of the therapist’s finger. In turn, the phrenic nerve stimulated the diaphragm (respiratory muscle). The output of the spinal roots of the phrenic nerve is located at the level of the 3–5 cervical vertebrae of the spine (C_3_-C5 ). It may be assumed that thanksto afferent (sensory) fibres, the sensory impulse enters thecervical segments of the spinal cord. Vojta assumed that the stimulus moves further from the cervical segments of the spinal cord through the roots of the accessory nerve(XI) to the medulla oblongata, since the roots of the cranial nerve also run there [27]. He also suggested that during stimulation of the breast zone, interoceptors of the internal organs of the pleural cavity and mediastinum transmit stimuli through the vagus nerve (X) up to the medulla oblongata [27]. Embryologically, the vagus nerve(X) innervates the dorsal mesentery of the oesophagus which forms the diaphragm branches. This means thatthis level (lumbar spine L-_1_L_3_) is sufficient to observe thefirst functional connections of the vagus nerve and thephrenic nerve which innervate the diaphragm [28].

Based on these conclusions, the authors of the study assumed that the stimulation of the breast zone can lead to the transmission of a sensory stimulus from the diaphragm area to the medulla oblongata, where sCPG is located. There is a hypothetical nerve “pathway” that can be used by the sensory impulse to enter the circuit of the sCPG neural network by means of afferent fibres. In response, the stimulated central pattern generator activates the rhythmic movements of the oral cavity muscles responsible for sucking motions by means of the cranial efferent nerve fibres (i.e. V, VII, IX, X, XII). In practice, this change is reflected by the variations and improvement of the rhythmicity as well as the regularity of NS and NS.

## Conclusion

The stimulation of the breast zone based on the Vojta method directly affects the central pattern generator, which improves the rhythmicity and regularity of both non-nutritive and nutritive sucking in premature newborns.
